# Antifungal action and induction of resistance by *Bacillus* sp. strain YYC 155 against *Colletotrichum fructicola* for control of anthracnose disease in *Camellia oleifera*

**DOI:** 10.3389/fmicb.2022.956642

**Published:** 2022-08-25

**Authors:** Aiting Zhou, Fang Wang, Jiabi Yin, Ruiqi Peng, Jia Deng, Dezhou Shen, Jianrong Wu, Xiaoyun Liu, Huancheng Ma

**Affiliations:** ^1^Key Laboratory of National Forestry and Grassland Administration on Biodiversity Conservation in Southwest China, Southwest Forestry University, Kunming, China; ^2^Key Laboratory of Microbial Diversity Research and Application of Hebei Province, School of Life Sciences, Hebei University, Baoding, China; ^3^Dehong Forestry and Grassland Bureau, Dehong, China; ^4^Key Laboratory of Forest Disaster Warning and Control in Universities of Yunnan Province, College of Biodiversity Conservation, Southwest Forestry University, Kunming, China

**Keywords:** *Camellia oleifera*, anthracnose, *Colletotrichum fructicola*, biological control, endophytic bacteria

## Abstract

Anthracnose disease caused by *Colletotrichum fructicola* is a serious disease that can afflict *Camellia oleifera*. Biological control is a rapidly growing approach for the management of plant diseases. In this study, we investigated the bio-control efficiency and the defense responses of an endophytic *Bacillus tequilensis* strain YYC 155, which was isolated from the root nodules of the *Crotalaria pallida* against anthracnose disease, caused by *C. fructicola* in *C. oleifera. B. tequilensis* YYC 155 exhibited significant inhibitory activity against anthracnose disease, caused by *C. fructicola* in *C. oleifera.* YYC 155 can secrete extracellular hydrolases, such as chitinase and β-1, 3-glucanase, which produce lipopeptides that are antimicrobial and forms strong biofilms. In addition, in treatment with YYC 155, the cell membranes of *C. fructicola* were injured and the leakage of cell contents from the mycelia of the pathogen was increased. Spraying 1 × 10^7^ cells mL^–1^ bacterial suspension of YYC 155 on *C. oleifera* leaves enhanced the activity of key enzymes in *C. oleifera* associated with the phenylpropanoid pathway and increased the content of phenolic compounds and flavonoids. Results of our study indicate that *B. tequilensis* YYC 155 may potentially represent an effective biocontrol agent against anthracnose disease in *C. oleifera*.

## Introduction

*Camellia oleifera* is an evergreen shrub in the *Camellia* family. It is known for its production of tea oil or *Camellia* oil and is an economically important crop in southern China (Wu et al., 2019). *C. oleifera* is an important component in many health care products due to the high content of unsaturated fatty acids in its oil and other nutrients (Zhang et al., 2012; Wu et al., 2019). The production of *C. camellia*, however, is hampered by anthracnose disease, caused by *Colletotrichum fructicola*, a serious disease that is widespread in *Camellia* production areas. It can infect various parts of *C. oleifera* during its entire production cycle, including dropped leaves and fruit. Anthracnose disease has led to a decline in *Camellia* production and results in huge economic losses every year (Perrett et al., 2003). Traditionally, anthracnose control has mainly been achieved using chemical fungicides. While these agents are effective in controlling anthracnose, the overuse of chemical pesticides may have negative effects on human health and the environment (Kilani-Feki et al., 2016). Therefore, the development of safe and effective biological control methods is attracting increasing public attention.

The use of a variety of different microbial antagonists as biocontrol agents appears to represent a promising alternative for controlling anthracnose (Shiomi et al., 2006). In this regard, endophytic microorganisms (bacteria and fungi) are being extensively studied for their potential as biocontrol agents due to their antagonistic activity against pathogens and their ability to induce resistance in host plants (Shiomi et al., 2006; Mahdi et al., 2014). The bacterial genus *Bacillus* has been identified as a frequently occurring endophyte in many plant species (Vendan et al., 2010). Cui et al. (2020) reported that *Bacillus velezensis* 8-4 isolated from potatoes exhibits a strong antagonistic ability against potato scab and increases potato yields. Li et al. (2009) screened 175 endophytic bacteria from *C. oleifera* to control the anthracnose caused by *Colletetrichum gloeosporioides* with confront culture method and *Bacillus licheniformis* YB128 had the best antagonistic effect with 86.1% inhibitory rate. An endophytic bacteria, *Bacillus subtilis* strain 1-L-29, isolated from *Camellia oleifera* could efficiently inhibit the *C*. *fructicola* growth and colonize the root surfaces of *C. oleifera* (Xu et al., 2020). It is known that *Bacillus* species produces a variety of compounds, including antibiotics (Leifertl et al., 1995), lipopeptides (Ahimou et al., 2000), cell wall-degrading enzymes (Leelasuphakul et al., 2006), and volatile organic compounds (VOCs) (Fiddaman and Rossall, 1993), which play a role in the inhibitory activity of *Bacillus* against many plant pathogens. To further explore the mechanisms of biocontrol endophytes, some researchers have focused on their effects on the physiological metabolism of pathogens. Dai et al. (2021) found that *Bacillus pumilus* HR10 was capable of injuring the cell membrane of pathogens and increasing the leakage of cell components. Additionally, antagonistic *Bacillus* species could induce systemic resistance of plants, especially the phenylpropanoid metabolism that plays a very important role in plant disease resistance.

Elicitation of plant defense mechanisms by these *Bacillus* strains has been demonstrated in root irrigation treatment and root-irrigation with *Bacillus* strains effectively increased plant defense-related enzyme activities (Chen et al., 2010; Zhang et al., 2022). Whether leaf spraying treatment of biological control agents have the same effects of inducing disease resistance as root irrigation treatment. Therefore, the objective of the present work was to investigate the impacts of foliar spray with YYC 155, which was previously isolated from the root nodule of *Crotalaria pallida* on the activity of some defense-related enzymes and compounds in the phenylpropanoid pathway of *C. oleifera* plants. Moreover, we assessed the ability of *Bacillus* sp. strain YYC 155 (subsequently identified as a strain of *B. tequilensis*) to inhibit the growth of *C. fructicola in vitro*, determined the presence of genes in YYC 155 responsible for the synthesis of antimicrobial compounds, and determined the effect of YYC 155 on parameters such as cell membrane integrity and cell leakage in *C. fructicola.*

## Materials and methods

### Plants, fungal inoculum, and bacterial strain

Two-year-old shrubs of *C. oleifera* (“Changlin No. 4”) were obtained from Yingjiang Linli Oil Tea Co., Ltd., in Dehong Prefecture, Yunnan Province of China, and then grown in a greenhouse with the following conditions 25°C and 85% relative humidity.

Pathogen isolation: Leaves of *C. oleifera* infected exhibiting anthracnose symptoms were collected from Dehong Prefecture, Yunnan Province of China. The leaves were brought to the laboratory and rinsed thoroughly with tap water. Five pieces (3–5 mm) of leaf tissue were taken from the border of lesions of 30 leaves and surface sterilized with 75% ethanol for 10 s and 0.1% mercuric chloride for 3 min. The leaf sections were then rinsed three times with sterile distilled water (SDW), dried on sterile filter paper, and placed on the Potato Dextrose Agar (PDA) medium amended with streptomycin sulfate (300 mg L^–1^) (w/v) and incubated for 1 week at 25°C. Pathogenicity of the collected *C. fructicola* isolates was confirmed on *C. oleifera* plants. The most aggressive isolate of *C. fructicola* was selected for use in the *in vitro* and *in vivo* bioassays.

Eight bacterial strains were isolated from root nodules of *Crotalaria pallida* in a previous study and maintained in a lysogeny broth (LB) medium at 4°C at the Key Laboratory of the National Forestry and Grassland Administration on Biodiversity Conservation in Southwest China, Southwest Forestry University, Kunming, Yunnan Province of China.

### Screening of bacterial strains *in vitro*

We conducted dual-culture assays to determine the antifungal capacity of the bacterial strains *in vitro* in Petri plates (diameter, 90 mm). Five microliters (OD600 = 0.1) droplets of each of the eight bacterial strains were then placed on Petri plates. Four droplets were placed around the edge of the Petri dish. An agar-mycelial plug (diameter, 6 mm) obtained from the margin of a *C. fructicola* culture during active growth was placed upside down in the center of the Petri plate center approximately 30 mm away from the droplets of the bacterial suspension. Three replicates of every bacterial strain were prepared, as well as pathogen-only plates (control). After inoculation, the plates were placed in an incubator at 25°C. The diameter of the colony of *C. fructicola* was recorded daily beginning at day 4 and ending at day 9 of incubation. Antifungal activity was calculated as the proportion of mycelial growth inhibition relative to the control using the formula, (R1–R2)/R1 × 100%. In the formula, R2 represents pathogen colony diameter in the presence of a bacterial isolate, while R1 is pathogen colony diameter in control plates.

### Effect of YYC 155 on *C. fructicola* in detached leaves of *C. oleifera*

The bacterial strain YYC 155, which had exhibited the best inhibitory activity against *C. fructicola in vitro* in the previous experiment, was used to assess its inhibitory effect on *C. fructicola* in detached leaves taken from healthy *C. oleifera* plants. The detached leaves were washed with sterilized distilled water (SDW), dried on paper towels, and placed abaxial side up in Petri dishes containing two filter papers saturated with SDW (Dorighello et al., 2015). Four artificial wounds were administered at the same position on each leaf using a sterile needle. Each wound was inoculated with 5 μL of a YYC 155 suspension at a concentration of 1 × 10^7^ cells mL^–1^ (previously cultured for 24 h). Subsequently, 24 h later YYC 155-inoculated leaves were inoculated with mycelial disks (diameter, 5 mm) of actively growing mycelia of *C. fructicola*. Wounded leaves inoculated with just *C. fructicola* and leaves inoculated with just SDW served as positive and negative controls, respectively. Three leaves with four wounds each were used for each treatment and the experiment was repeated twice. Lesion diameters on leaves were measured daily beginning on the 3rd day after pathogen inoculation. The inhibition of *C. fructicola* was determined by comparing the diameter of the lesion.

### Identification of bacterial strain YYC 155

Genomic DNA was extracted from bacterial strain YYC 155 using an Ezup Column Bacteria Genomic DNA Purification Kit (Sangon Biotech, Shanghai, China), according to the manufacturer’s instructions. We utilized the universal primers 27F (5′-AGAGTTTGATCMTGGCTCAG-3′) and 1492R (5′-TACGGYTACCTTGTTACGACTT-3′) for PCR amplification of the 16S rRNA gene (Fredriksson et al., 2013). The obtained PCR products were purified and sequenced. The consensus 16S rRNA sequence was analyzed at https://www.ezbiocloud.net, to identify phylogenetic neighbors and to calculate the pairwise similarity of the 16S rRNA gene sequence. CLUSTALW in Mega 7.0 was used to conduct a similarity calculation and multiple alignments (Kumar et al., 2016). A 16S rRNA phylogenetic tree was constructed using the neighbor-joining method with 1,000 bootstrap replicates.

### Effect of YYC 155 on membrane permeability and cell leakage in *C. fructicola*

Relative electrical conductivity was measured as described by Elsherbiny et al. (2021) with slight modification. An agar-mycelial plug (6 mm diameter) taken from the edge of an actively growing culture of *C. fructicola* was placed in a 50 mL centrifuge tube containing 20 mL of Potato Dextrose Broth (PDB) and incubated for 72 h at 25°C on a rotary shaker set at 160 rpm/min. The resulting mycelia were then washed 3 × with SDW and transferred to a 10 mL centrifuge tube containing 4 mL of a YYC 155 suspension at 1 × 10^7^ cells mL^–1^. Tubes containing SDW instead of YYC 155 served as a control. The mixtures were incubated at 28°C on a rotary shaker set at 180 rpm/min, and samples were collected at 24 h, 48 h, 72 h, 96 h, and 120 h. The electrical conductivity of the mixtures was determined using an electrical conductivity meter (DDS-307, China), after which the mixtures were heated in a boiling water bath for 15 min. After cooling to room temperature, the final conductivity measurement was taken. Relative conductivity was determined using the formula, relative conductivity (%) = initial conductivity/final conductivity × 100%. Each assay was conducted in triplicate.

The assessment of injury to *C. fructicola* used the same experimental design used to measure electrical conductivity; however, only the supernatant was used in the assessment. The cultures were incubated at 24 h, 48 h, 72 h, 96 h, and 120 h, and then centrifuged at 5,000 *g*/min for 2 min at 4°C. The supernatant was used to assess the leakage of extracellular nucleic acids and soluble protein content, while the remaining mycelia were used to measure malondialdehyde (MDA) content (Dai et al., 2021). The values for leakage of extracellular nucleic acids are expressed as the OD_260_ mL^–1^, the values for soluble protein content are expressed as milligram per mL of supernatant, and the values for MDA are expressed as nmol per gram of mycelia. Each assessment had three replicates and the experiment was conducted twice.

### Scanning electron microscopy analysis

To test the effect of YYC 155 on fungal mycelia morphology, a scanning electron microscope (SEM) was utilized. Mycelial plugs of *C. fructicola* with 6-mm-diameter were obtained from the interaction region treated with strain YYC 155 through dual-culture assays according to the above-mentioned method in section “Screening of bacterial strains *in vitro*” and mycelial plugs of the *C. fructicola* without strain YYC 155 were as control. The mycelial plugs were fixed with 2.5% glutaraldehyde at 4°C overnight, rinsed with 0.1 M phosphate buffer three times, and dehydrated through a series gradient concentration of ethanol followed by drying in a critical point drainer by liquid CO_2_. Then the samples were coated with gold and observed using a Hitachi Regulus 8100 Scanning Electron Microscope.

### Biofilm formation

The ability of YYC 155 to form a biofilm was assessed as previously described (Banerjee et al., 2018) with some modifications. A single colony of YYC 155 obtained from a 24 h culture of YYC 155 growing on LB agar plates at 28°C was placed in LB liquid medium and cultured for 24 h at 28°C on a rotary shaker set at 160 rpm/min. The cell density of the 24 h culture was adjusted to 1 × 10^7^ cells mL^–1^, and 1,500 μL of aliquots were placed into individual wells of a 24-well polystyrene plate that contained 1,000 μL LB medium. Three replicate wells were used to assess biofilm formation at different timepoints. Three wells containing LB medium alone were used as a control. The plates were incubated at 28°C for 12 h, 24 h, 48 h, 72 h, 96 h, and 120 h. At each timepoint, the medium with the pathogen was emptied from the wells and the adherent biofilm was stained with the 0.1% aqueous crystal violet solution for 10 min. The wells were then rinsed 3 × with SDW and the biofilms were dissolved by adding 40% acetic acid solution (2,500 μL) to each well for 5 min. The absorbance of the acetic acid solution was then measured at OD_560_ to assess the ability of YYC155 to form a biofilm. Three duplicate wells were used for the control and bacteria at each time point, and the assay was conducted twice.

### Extracellular chitinase and β-1, 3-glucanase activity of strain YYC 155

The secretion of extracellular hydrolases by strain YYC 155 was assessed. The bacteria were grown in LB medium for 24 h at 28°C, and a single colony of YYC 155 was then transferred to LB liquid medium and cultured at 28°C on a rotary shaker set at 180 rpm/min. Chitinase and β-1, 3-glucanase activity in the culture medium was evaluated after 12 h, 24 h, 48 h, 72 h, 96 h, and 120 h of incubation. The enzymatic assays were conducted as described by Zhang et al. (2017) with some modifications. Chitinase activity is expressed in units, where one unit is defined as the enzyme activity catalyzing the formation of 1 mg of *N-*acetyl-D-glucosamine per milliliter of culture medium per hour. B -1, 3-glucanase is also denoted in units, where one unit is defined as the amount of enzyme needed to produce 1 mg of reducing sugar per milliliter of culture suspension. Three replicates were measured in each experiment and the experiment was repeated twice.

### PCR amplification of lipopeptide biosynthetic genes in *B. tequilensis* YYC 155

Genomic DNA was isolated from *B. tequilensis* YYC 155 as described in section “Identification of bacterial strain YYC 155.” Lipopeptide biosynthetic genes were then amplified using the genomic DNA as a template. PCR amplification was conducted in 50 μL reaction volume in a thermocycler using the following protocol: initial denaturation at 95°C for 3 min, 32 cycles of 94°C for 30 s, annealing temperature varied depending upon the specific primers, and extension at 72°C for 40 s. The annealing temperature and the primers used in the PCR amplification are listed in Supplementary Table 1.

### Effect *B. tequilensis* YYC 155 on defense-related enzyme activity and compounds in *C. oleifera*

A total of 20 mL of a bacterial suspension of YYC 155 at the concentration of 1 × 10^7^ cells mL^–1^, previously cultured at 28°C on a rotary shaker set at 180 rpm/min for 24 h, was evenly sprayed on the leaves of 2-year-old potted shrubs of *C. oleifera*, with the application of sterile distilled water treatment serving as a control. The effect of the application of s YYC 155 on the level of total phenols, flavonoids, and the activity of phenylalanine ammonia lyase (PAL), polyphenol oxidase (PPO), cinnamic acid-4-hydroxylase (C4H), 4-coumarate: CoA ligase (4CL), and chalcone isomerase (CHI) were assessed in leaves of *C. oleifera* collected at 1 d (24 h after the application of YYC 155), 5 d, 10 d, 15 d, 20 d, 25 d, and 30 d. The level of total phenols and flavonoids was measured using the method of Wang et al. (2018) in which absorbance was measured at 280 nm or 325 nm. Content is expressed on a fresh weight basis as OD280 g^–1^ and OD325 g^–1^, respectively. PAL, C4H, 4CL, and CHI activities were also calculated using the procedure described by Wang et al. (2018). PPO was assayed according to Wang et al. (2021). Results are expressed as units per gram fresh weight of *C. oleifera* leaves.

### Statistical analysis

A completely randomized design was used in the experiments. The data were statistically analyzed using Duncan’s multiple range test or an independent *t*-test in SPSS20.0. Statistical significance was set at *P* < 0.05.

## Results

### Screening bacterial strains for antagonistic activity *in vitro*

**FIGURE 1:**
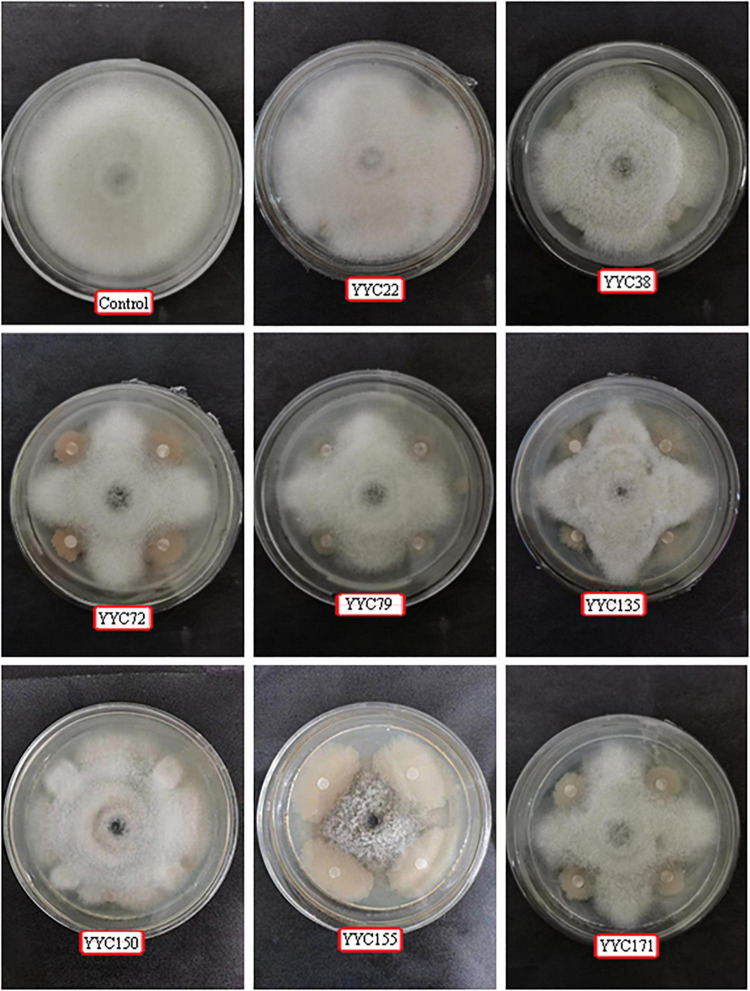
Representative photos illustrating the antagonistic activity of eight bacterial strains isolated from root nodules of *C. pallida* against *C. fructicola in vitro.* Three independent replicates were used for different strains.

**FIGURE 2:**
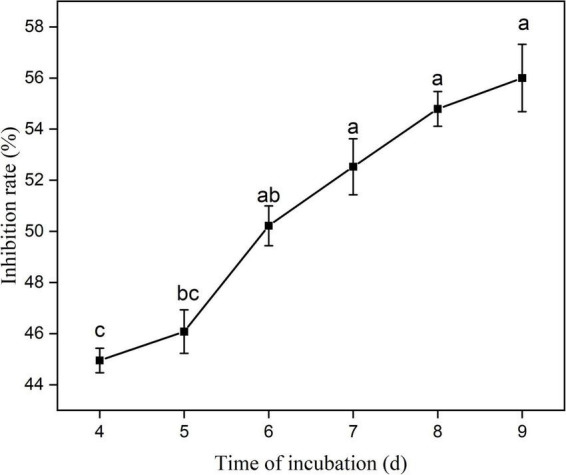
Percentage inhibition of *C. fructicola* by *Bacillus tequilensis* strain YYC 155 *in vitro* during 9 days of co-culture. Three duplicates are used for control and strain YYC 155 at each time point. Data represent the mean ± standard error (se). Different letters indicate a significant difference between the inhibitor rate on different days as determined by Duncan’s multiple range tests (*P* < 0.05).

The antifungal activity of eight strains of bacteria isolated from root nodules of *C. pallida* was assessed in a dual culture plate assay. The eight strains (YYC 22, YYC 38, YYC 72, YYC 79, YYC 135, YYC 150, YYC 155, and YYC 171) exhibited varying levels of inhibition of *C. fructicola*, relative to the control, after 9 d of co-culture. The percentage of inhibition of the different strains was 18.02%, 33.72%, 43.80%, 33.91%, 45.35%, 21.51%, 56.00%, and 34.88%, respectively (Figure 1). Strain YYC 155 exhibited the greatest level of inhibitory activity against the mycelial growth of *C. fructicola* and its inhibition level increased with incubation time (Figures 1, 2). Based on these results, strain YYC 155 was used in the subsequent experiments.

### Inhibitory activity of YYC 155 against *C. fructicola* on detached leaves of *C. oleifera*

**FIGURE 3:**
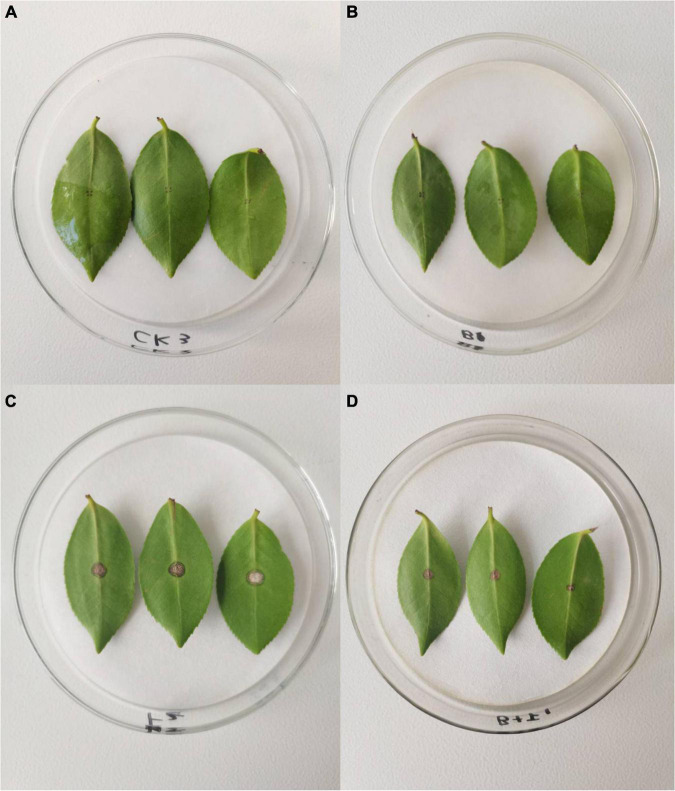
The effect of *Bacillus tequilensis* YYC 155 on lesion development in detached leaves of *C. oleifera* caused by *C. fructicola* at 5 days after pathogen inoculation. Three independent replicates were used for different treatments and three detached leaves were used for each replicate. **(A)** Sterile water treatment (control), **(B)** YYC 155 treatment, **(C)** *C. fructicola* treatment, **(D)** YYC 155 + *C. fructicola* treatment.

Figure 3 demonstrates that wounded, detached leaves of *C. oleifera* treated with strain YYC 155 exhibited a decreased rate of lesion development. Lesion diameter in *C. oleifera* leaves treated with YYC 155 was significantly (*P* < 0.05) smaller than it was in leaves inoculated with *C. fructicola* alone beginning from the 3rd day after pathogen inoculation (Table 1). No evidence of lesions was observed on leaves treated only with sterile water or only with YYC 155, the latter indicating that strain YYC 155 is not a plant pathogen of *C. oleifera*.

**TABLE 1:** Lesion diameter caused by *C. fructicola* on detached leaves of *C. oleifera.*

Time (d)	Sterile water (mm)	YYC 155 (mm)	*C. fructicola* (mm)	YYC 155 + *C. fructicola* (mm)
3	0.0 ± 0.0c	0.0 ± 0.0c	4.0 ± 0.2a	2.4 ± 0.3b
5	0.0 ± 0.0c	0.0 ± 0.0c	5.8 ± 1.5a	2.9 ± 0.1b
7	0.0 ± 0.0c	0.0 ± 0.0c	8.9 ± 1.4a	4.0 ± 0.1b
9	0.0 ± 0.0c	0.0 ± 0.0c	12.2 ± 1.7a	5.0 ± 0.2b

Leaf treatments were sterile water, YYC 155, C. fructicola, and YYC 155 followed by C. *fructicola*.

Three duplicates are used for different treatments at each time point and three detached leaves were used for each replicate.

Data represent the mean ± standard error (se).

Different letters indicate a significant difference between treatment groups on the designated day as determined by Duncan’s multiple range test (*P* < 0.05).

### Taxonomic classification of bacterial strain YYC 155

**FIGURE 4:**
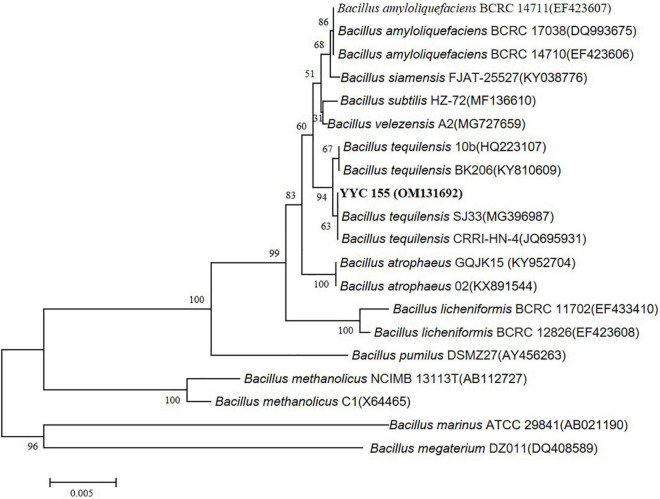
Phylogenetic tree of YYC 155 based on the 16S rRNA gene sequence. The tree was constructed using the neighbor-joining method with 1,000 bootstrap replicates. Bootstraps greater than 50% are indicated. Bars = 0.005 nucleotide substitutions per site.

We amplified the 16S rRNA gene sequence of YYC 155 from genomic DNA to determine its taxonomic classification. Then, the nearly complete 16S rRNA gene sequence of 1,452 bp was amplified and sequenced. Nearly, the entire 1,452 bp sequence of the 16S rRNA gene was amplified and sequenced. Sequence analysis indicated that YYC 155 appears to be *B. tequilensis* having 100% homology to *B. tequilensis* strains CRRI-HN-4 and SJ33. The phylogenetic tree of the sequenced 16S rRNA also indicated that it is in the same clade as *B. tequilensis*, further suggesting that YYC 155 is a strain of *B. tequilensis* (Figure 4). The 16S rRNA sequence of YYC 155 was deposited at DDBJ/ENA/GenBank (accession number, OM131692).

### Effect of YYC 155 on cell membrane permeability and content of cellular compounds in *C. fructicola*

**FIGURE 5:**
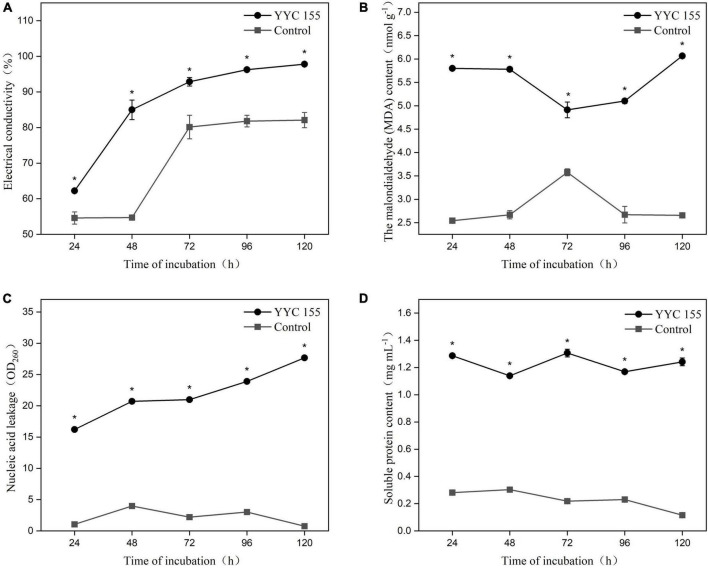
Effect of strain YYC 155 on cell membrane permeability and the content of cellular compounds in *C. fructicola.* **(A)** Electrical conductivity; **(B)** the malondialdehyde (MDA) content; **(C)** nucleic acid leakage; **(D)** soluble protein content. Three duplicates are used for the control and YYC 155 treatment against *C. fructicola* at each time point and were measured three times for each replicate. Data represent the mean ± standard error (se). Asterisks indicate a significant difference between the control and YYC 155 treatment groups at the designated time point as determined by the *t*-test (*P* < 0.05).

The permeability of the cell membrane of *C. fructicola* was assessed as the relative electrical conductivity of mycelia (Figure 5A). The electrical conductivity of *C. fructicola* mycelia co-incubated with YYC 155 increased significantly over the entire course of co-incubation relative to the control treatment.

Lipid peroxidation in *C. fructicola* mycelia was assessed by measuring malondialdehyde (MDA), a marker of oxidative stress injury. The MDA level in *C. fructicola* mycelia co-cultured with YYC 155 was significantly higher (*P* < 0.05), relative to the control mycelia. The highest level of MDA (6.07 ± 0.05 nmol g^–1^) in *C. fructicola* co-cultured with YYC 155 was observed after 120 h of co-incubation (Figure 5B). These results indicate that exposure of *C. fructicola* to YYC 155 induces injury to the cell membranes of mycelia of *C. fructicola* and as a result increases the permeability of the cell membrane, as evidenced by the increased level of electrolyte leakage.

The effect of YYC 155 on the leakage of nucleic acids and protein from the mycelia of *C. fructicola* is also an indicator of cell membrane damage. The level of leaked nucleic acids and soluble proteins present in the growth medium was significantly higher (*P* < 0.05) in the YYC 155 treatment group than in the control during the entire time of co-incubation (Figures 5C,D).

### Scanning electron microscope

**FIGURE 6:**
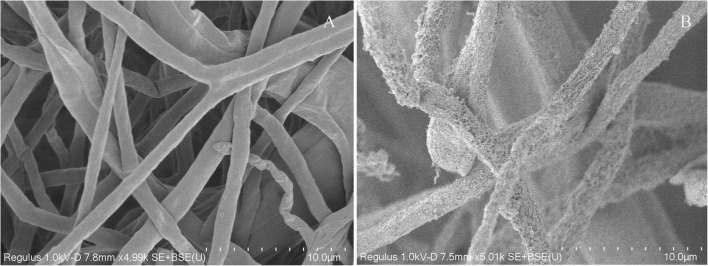
The morphological changes of *C. fructicola* hyphae under SEM. **(A)** Untreated hyphae of *C. fructicola* (control). **(B)** Hyphae of *C. fructicola* treated with strain YYC 155.

Scanning electron microscope analysis showed that *C. fructicola* hyphae changed significantly under the action of strain YYC 155 compared with the control (Figure 6). Hyphae were round and full in the control, with complete morphology and structure, uniform thickness, and smooth surface, whereas the hyphae of *C. fructicola* affected by strain YYC 155 were seriously damaged, with surface shrinkage and leakage of contents.

### Biofilm formation by YYC 155

**FIGURE 7:**
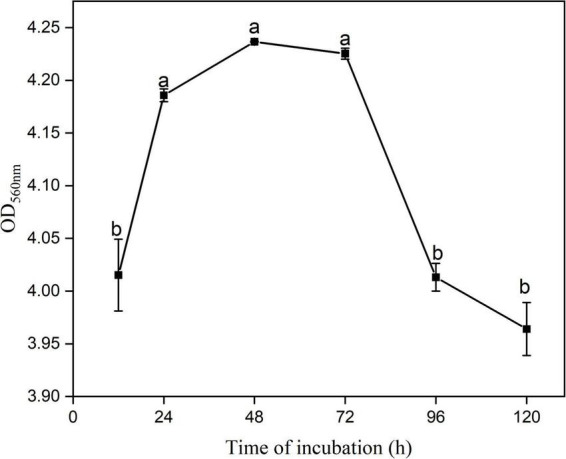
The ability of *B. tequilensis* YYC 155 to form a biofilm at 28°C. Three duplicate wells are used for YYC 155 at each time point. Data represent the mean ± standard error (se). Different letters represent significant differences in biofilm-forming capacity at the designated timepoint as determined by Duncan’s multiple range test (*P* < 0.05).

*B. tequilensis* YYC 155 exhibited the ability to form biofilms on good surfaces of polystyrene plates after 12 h of incubation at 28°C (Figure 7). The data suggest that the bacteria undergo a period of initial adhesion, film-formation, and then a steady dissociation at 12–24 h, 24–72 h, and 72–120 h, respectively. Notably, biofilm formation was significantly higher (*P* < 0.05) at 24–72 h than at the other time points. These results indicate that YYC 155 cultured for 24–72 h has a strong ability to form a biofilm, which contributes to its successful colonization of plant surfaces, both external and internal.

### Extracellular chitinase and β-1, 3-glucanase activity by strain YYC 155

**FIGURE 8:**
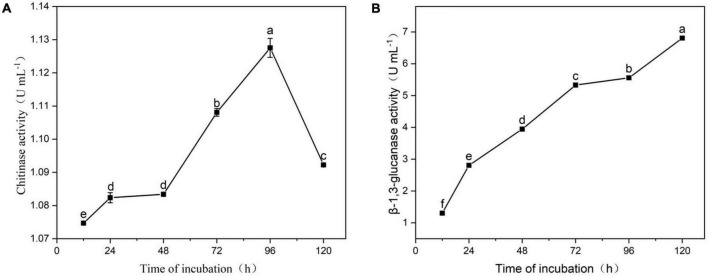
Extracellular chitinase **(A)** and β-1, 3-glucanase **(B)** activity by strain YYC 155. Three duplicates were measured for YYC 155 at each time point and were measured three times for each replicate. Data represent the mean ± standard error (se). Different letters represent a significant difference in enzyme activity between the different timepoints as determined by Duncan’s multiple range test (*P* < 0.05).

Figure 8A indicates that strain YYC 155 can secrete defense-related chitinase extracellularly. Chitinase enzyme activity reached its highest level at 96 h (1.13 ± 0.0029 U mL^–1^), then decreased sharply. Figure 8B indicates that strain YYC 155 can secrete β-1, 3-glucanase enzyme extracellularly, and that there was a linear increase in activity over the course of incubation. YYC 155 maintained a high level of extracellular β-1, 3-glucanase activity (6.81 ± 0.0016 U mL^–1^) up to 120 h of incubation.

### Lipopeptide biosynthesis genes in strain YYC 155

**FIGURE 9:**
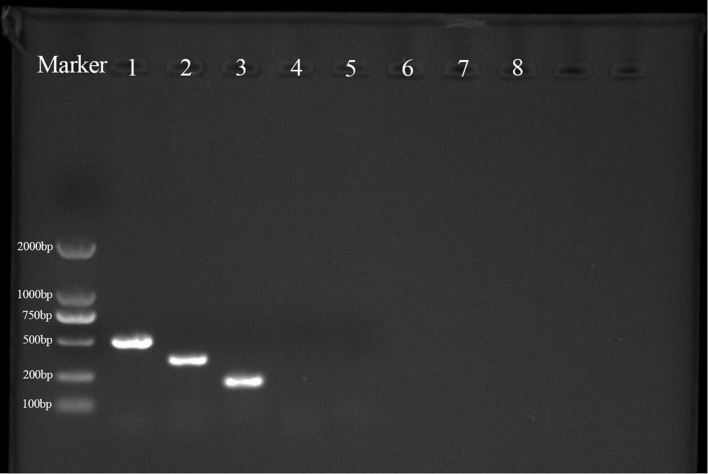
PCR products were obtained from the amplification of lipopeptide biosynthetic genes in strain YYC 155. M is a size marker; 1–8 are PCR products for genes encoding *bacA*, *ppsD*, *srfAA*, *fenD*, *baC*, *baE*, *bmyB*, and *ituB*, respectively. Only primers for the first three genes produced a PCR product.

PCR products for the lipopeptide synthesis genes *bacA* (500 bp), *ppsD* (350 bp), and *srfAA* (200 bp), which encode bacylisin, plipastatin, and surfacing, respectively, were obtained from strain YYC 155 (Figure 9). PCR products for *baC*, *baE*, *bmyB*, and *ituB* were not obtained.

### Effect of YYC 155 on defense-related compounds and enzyme activity in *C. oleifera*

**FIGURE 10:**
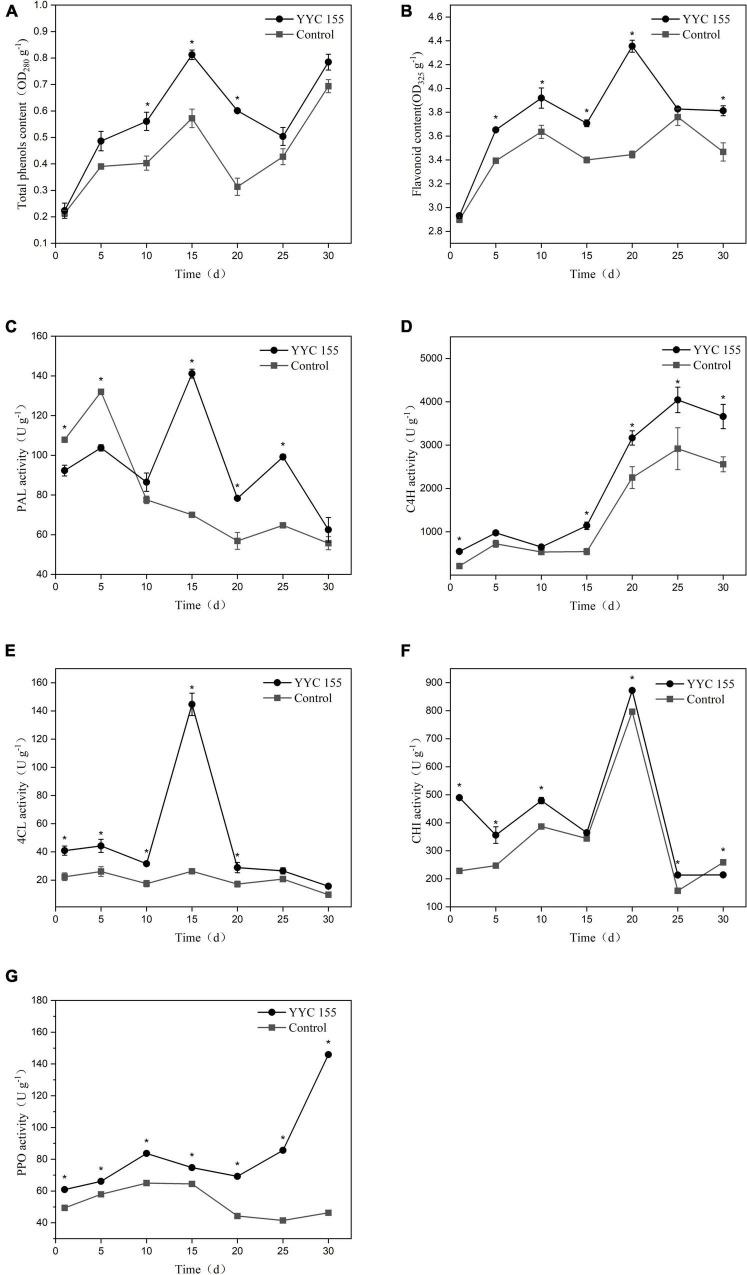
Effect of *B. tequilensis* YYC 155 on the level of total phenols **(A)** and flavonoids **(B)**, and the activity of PAL **(C)**, C4H **(D)**, 4CL **(E)**, CHI **(F)**, and PPO **(G)** in *C. oleifera*. Three duplicates are used for the control and YYC 155 treatment *C. oleifera* leaves at each time point and were measured three times for each replicate. Data represent the mean ± standard error (se). Asterisks represent a significant difference between the two treatment groups at the designated timepoint as determined by the *t*-test (*P* < 0.05).

The level of total phenols exhibited a similar pattern in C. *oleifera* leaves in both the YYC 155 treatment group and the control over the entire period of assessment (Figure 10A). *C*. *oleifera* treated with *B. tequilensis* YYC 155, however, had a higher level of total phenols, relative to the control, which increased significantly over the measured time period and peaked at the 15th day, and then subsequently decreased from day 15–25. The YYC 155 treatment group had a significantly higher (*P* < 0.05) level of total phenols, relative to the control, on days 10–20. The content of flavonoids in the YYC 155 treatment group was also higher than it was in the control group (Figure 10B). Flavonoid content exhibited an increasing pattern in both the control and the YYC 155 treatment group; however, the increase was very dramatic in the latter group, reaching its highest level at 20 days after treatment, after which, the levels exhibited a continuous decrease. Significantly higher levels (*P* < 0.05) of flavonoids were observed in the YYC 155 treatment group, relative to the control, at all timepoints except days 1 and 25. As indicated in Figure 10C, PAL activity in the YYC 155 treatment group exhibited an “M” pattern, peaking on day 15. PAL activity in the YYC 155 treatment group was significantly higher (*P* < 0.05), relative to the control throughout the assessment period, except at the initial stage. C4H activity in the two treatment groups exhibited a similar pattern of variation, steadily increasing up to day 25 and then decreasing from day 25 to day 30. A significantly higher (*P* < 0.05) level of C4H activity was exhibited in the YYC 155 treatment group, relative to the control, except on day 5 and day 10 (Figure 10D). In correspondence with Figure 10E, 4CL activity in the YYC 155 treatment group was mostly significantly higher (*P* < 0.05), relative to the control, reaching a maximum level of activity at day 15. CHI activity in the YYC 155 treatment group had a significantly higher (*P* < 0.05) level of activity, relative to the control, during the first 20 days, reaching a maximum of activity, after which CHI activity decreased (Figure 10F). Significant differences were not observed, however, on day 15. Lastly, the YYC 155 treatment group exhibited enhanced PPO activity, relative to the control, beginning on day 1 (Figure 10G) and was significantly higher (*P* < 0.05), relative to the control during the entire period of assessment (30 days).

## Discussion

In recent years, the use of plant-associated endophytes for biological control has received a great deal of attention and ongoing research efforts (Hong and Park, 2016). In this study, strain YYC 155, an endophytic bacterium isolated from root nodules of *Crotalaria pallida*, was identified as *B. tequilensis* by 16S rRNA gene sequencing. *B. tequilensis* YYC 155 grew faster than other isolated strains, and effectively inhibit the mycelial growth of *C. fructicola in vitro*, and also inhibited the development of anthracnose lesions caused by *C. fructicola* on detached leaves of *C. oleifera*, an economically important shrub that serves as a source of seed oil (Figures 1, 3). The inhibitory activity of strain YYC 155 may potentially be attributed to its ability to successfully compete for nutrients and space with pathogens (Liu et al., 2013).

Biological agents also have direct or indirect impacts on the cell membrane of fungi, affecting both membrane permeability and structure, resulting in increased leakage of critical cellular components, such as nucleic acids and proteins (Elsherbiny et al., 2021). Relative conductivity, MDA content, as well as nucleic acid and soluble protein leakage are often used to determine the degree of cell membrane damage (Dai et al., 2021; Elsherbiny et al., 2021). We demonstrated that exposure to *B. tequilensis* YYC 155 significantly increased relative conductivity and MDA levels in the anthracnose pathogen, *C. fructicola*, thus increasing membrane permeability, and providing evidence of membrane structural damage (Figure 5). Alterations in membrane structure increase membrane permeability and result in the release of proteins and nucleic acids outside the cell. We observed significant differences in membrane permeability and the leakage of cellular contents in the mycelia of *C. fructicola* exposed to *B. tequilensis* YYC 155, relative to the control, throughout the entire period of co-incubation, indicating that the cell membrane of *C. fructicola* was damaged by YYC 155. YYC 155 could affect the cell integrity and physiological metabolism of the pathogen, thereby affecting the growth of pathogens to achieve the purpose of inhibiting pathogens.

Several studies have demonstrated that *Bacillus* species can produce multiple types of antimicrobial compounds that inhibit the growth of pathogens and negatively affect their metabolism (Stein, 2005; Fan et al., 2017; Guardado-Valdivia et al., 2018). Antimicrobial substances produced by *Bacillus* spp. are mainly divided into two categories, ribosome-synthesized peptides, such as bacteriocin, and small antimicrobial peptides enzymatically synthesized in non-ribosomal pathways, which primarily comprise cyclic lipopeptides (CLPs) (Romero et al., 2007). Lipopeptides are an important *Bacillius* metabolite, and comprise three major families, iturins, fengycins, and surfactins (Liu et al., 2020). In this study, genes involved in the synthesis of lipopeptides were detected in YYC 155 by PCR amplification, including *bacA*, *ppsD*, and *srfAA*, which function in the synthesis of bacylisin, plipastatin, and surfactin, respectively (Figure 9). Lipopeptides, such as surfactin and other CLPs, produced by antagonistic microorganisms, not only have antimicrobial properties, but have also been reported to act as elicitors that induce disease resistance responses in plants (Ongena et al., 2007; Gond et al., 2015). *B. tequilensis* has also been reported to inhibit diverse fungal pathogens through the activity of antifungal enzymes (Defilippi et al., 2018). Therefore, their production by bacterial biocontrol agents should be investigated as a mechanism of action. Antifungal enzymes, such as chitinase and β-1, 3-glucanase, are known to inhibit the growth of many pathogens and have the ability to disrupt and degrade fungal cell walls (Aktuganov et al., 2008; Zhang et al., 2017). We demonstrated that *B. tequilensis* YYC 155 strain secreted chitinase and β-1, 3-glucanase and that these enzymes had a high level of activity (Figure 8), which consequently destroyed the cell wall of pathogenic fungi *C. fructicola*, as observed under SEM analysis (Figure 6).

In addition to antimicrobial compounds and antifungal enzymes, other features of strain YYC 155 are also likely contributed to its biocontrol activity. Therefore, we also examined biofilm formation. Biofilms are defined as a group of organized bacteria surrounded by a self-produced matrix of extracellular polymeric substances, that allow the colonies to adhere to the surface of both living and inanimate objects (Mah et al., 2003; Dai et al., 2021). Bacteria can adhere to plant tissues, hyphae, and spores, and form a biofilm. The biofilm allows them to compete against fungal pathogens, interfering with their ability to acquire nutrients from the host and blocking their ability to occupy space or receive developmental signals from plant host tissues (Liu et al., 2013). Our assay confirmed that *B. tequilensis* YYC 155 exhibited a strong ability to form a biofilm *in vitro* when cultured in LB liquid (Figure 7). The ability to form biofilms also increases their ability to colonize plants and provide protection against infection by pathogens (Haggag and Timmusk, 2008). When biocontrol agents pre-colonize host plants, antifungal substances are produced and are present at a level that protects plants to varying degrees from pathogen infection (Hang et al., 2005; Chen et al., 2020).

Previous studies have demonstrated that antagonistic microorganisms can both inhibit fungal pathogens and produce elicitors that activate defense responses in plants and enhance disease resistance (Chen et al., 2020). In this regard, the phenylpropanoid pathway represents a major pathway for the synthesis of plant secondary metabolites that are associated with plant disease resistance (Xu et al., 2019). PAL, C4H, 4CL, and CHI are important enzymes in the phenylpropanoid pathway. Plants produce numerous pathogen compounds *via* the phenylpropanoid pathway, such as flavonoids and phenolic substances (Chen et al., 2006). PPO is responsible for oxidizing phenolic substances while producing toxic quinines that restrict and induce mortality in fungal pathogens (Wang et al., 2021). The results of this study indicated that the activity of these defense-related enzymes in the phenylpropanoid pathway was maintained at a significantly higher level in *C. oleifera* after the plant host was sprayed with *B. tequilensis* YYC 155 on leaves. This induction of defense-related enzymes may have contributed to the reduced lesion development observed in YYC 155 treated leaves. Our results suggest that the enhancement of defense-related enzymatic activity in *C. oleifera* by foliar spray with *B. tequilensis* YYC 155 potentially contributes to increased disease resistance to anthracnose in *C. oleifera.* Foliar spray with *B. tequilensis* YYC 155 also elevated the level of both flavonoids and total phenols in *C. oleifera*, relative to the controls, over the entire course of the assessment period (30 days). Such results showed that the endophytic *B. tequilensis* strain YYC 155 might colonize the plant phylloplane and enter the plant leaves through the leaf stomata, thus improving plant-induced disease resistance. However, further studies are needed to investigate the interaction of endophytic *B. tequilensis* strain YYC 155 with the plant leaf surface.

## Conclusion

Collectively, the results of this study demonstrated that *B. tequilensis* YYC 155 inhibited anthracnose development in leaves of *C. oleifera* by inhibiting the growth of *C. fructicola.* The mechanism of control by *B. tequilensis* YYC 155 was associated with a direct antifungal effect on *C. fructicola* and the induction of enhanced resistance in *C. oleifera*. It appears that *B. tequilensis* YYC 155 targets the cell membrane of *C. fructicola*, increasing membrane permeability and inducing the leakage of cellular compounds, which together inhibit the growth of *C. fructicola*. Foliar spray with *B. tequilensis* YYC 155 also enhanced defense response in *C. oleifera* by enhancing the activity of defense-related enzymes that function in the phenylpropanoid pathway, and also promoted the accumulation of flavonoids and phenols. Thus, *B. tequilensis* YYC 155 may potentially represent an effective biocontrol agent against the anthracnose pathogen, *C. fructicola*, in the oil seed shrub, *C. oleifera.*

## Data availability statement

The datasets presented in this study can be found in online repositories. The names of the repository/repositories and accession number(s) can be found in the article/Supplementary material.

## Author contributions

AZ: formal analysis, data curation, visualization, validation, and writing—original draft. FW: conceptualization, supervision, data curation, and writing—review and editing. JY, DS, and JW: methodology and resources. RP: data curation and visualization. JD: methodology and data curation. XL: methodology and formal analysis. HM: project administration, funding acquisition, and writing—review and editing. All authors contributed to the article and approved the submitted version.
