# Supplementary material for: Antifungal action and induction of resistance by Bacillus sp. strain YYC 155 against Colletotrichum fructicola for control of anthracnose disease in Camellia oleifera
Source: Front Microbiol. 2022 Aug 25;13:956642. doi: 10.3389/fmicb.2022.956642 (PMC9453557; doi:10.3389/fmicb.2022.956642)
Supplement: Supplementary file 1 [file Table_1.DOC]

Table. S1. The primers for lipopetide biosynthetic genes

| Product | Target gene | Annealing Temp (ºC) | | Primer sequence (5’-3’) | Product size (bp) |
| --- | --- | --- | --- | --- | --- |
| Iturin | *ituB* | 55.1 | | ATCACCGATTCGATTTCA GCTCGCTCCATATTATTTC | 708 |
| Surfactin | *srfAA* | 55.8 | | TCGGGACAGGAAGACATCAT CCACTCAAACGGATAATCCTGA | 201 |
| Bacillomycin | *bmyB* | 55.3 | | CGAAACGACGGTATGAAT TCTGCCGTTCCTTATCTC | 371 |
| Fengycin | *fenD* | 57.6 | | TCAGCCGGTCTGTTGAAG TCCTGCAGAAGGAGAAGT | 231 |
| Bacillaene | *baE* | 57.6 | | CTCCGAAAGACGCAGAAT ACCGACTTTATCCGCTCC | 599 |
| Plipastatin | *ppsD* | 55.8 | | TTTTCTGCCCCCAGTACT AAATTGAATCGGTCATCCG | 346 |
| Bacillibactin | *baC* | | 57.6 | ATCTTTATGGCGGCAGTC ATACGGCTTACAGGCGAG | 595 |
| Bacylisin | *bacA* | | 55.8 | CAGCTCATGGGAATGCTTTT CTCGGTCCTGAAGGGACAAG | 498 |
